# From Algorithms to Biomarkers: Toward Personalized Management of Venous Thromboembolism

**DOI:** 10.3390/jcm14176172

**Published:** 2025-09-01

**Authors:** Andrea Boccatonda

**Affiliations:** Diagnostic and Therapeutic Interventional Ultrasound Unit, IRCCS Azienda Ospedaliero-Universitaria di Bologna, 40138 Bologna, Italy; andrea.boccatonda2@unibo.it or andrea.boccatonda@aosp.bo.it

Pulmonary embolism (PE) and venous thromboembolism (VTE) remain major contributors to global morbidity and mortality, yet their management has evolved significantly in recent years. Diagnostic efficiency has improved through clinical probability-based approaches, which combine Wells or Geneva scores with age-adjusted or probability-adapted D-dimer thresholds, which reduce unnecessary CT pulmonary angiography while maintaining safety [1,2]. More recently, strategies such as YEARS and 4PEPS have further refined diagnostic pathways, although they require broad external validation [2].

Treatment has been transformed by the widespread adoption of direct oral anticoagulants (DOACs), which are now first-line treatments for both initial and extended therapy. Reduced-dose regimens of apixaban or rivaroxaban have demonstrated superiority over aspirin in preventing recurrent VTE [3,4]. The American Society of Hematology (ASH) recommends indefinite anticoagulation for most cases of unprovoked VTE [5]. For low-risk PE, outpatient care is now supported by randomized trials and real-world studies, improving both patient convenience and healthcare resource use [6].

In intermediate-risk PE, current practice favors therapeutic anticoagulation with close monitoring, reserving reperfusion for clinical deterioration or selected intermediate-high risk patients with low bleeding risk [7]. Routine systemic thrombolysis is not recommended because, while it reduces hemodynamic decompensation, it increases major and intracranial bleeding (PEITHO) [8]. Interest has shifted to catheter-based strategies (ultrasound-facilitated low-dose lysis or mechanical thrombectomy), but definitive evidence vs. anticoagulation alone is still pending; pivotal trials are underway (HI-PEITHO and PE-TRACT) [9,10]. The PEERLESS trial demonstrated that large-bore mechanical thrombectomy reduced clinical deterioration and ICU admission compared with catheter-directed thrombolysis, though major bleeding rates were similar [11]. However, randomized comparisons with anticoagulation alone remain lacking, leaving uncertainty about which patients truly benefit from invasive strategies. Multidisciplinary decision-making is encouraged to individualize escalation based on RV dysfunction, biomarker profile, comorbidity, and bleeding risk.

System-level responses have emerged in the form of pulmonary embolism response teams (PERTs), designed to improve multidisciplinary decision-making and facilitate access to advanced therapy [12]. A retrospective study found that patients transferred after PERT activation, though more severely ill according to ESC classification, were more likely to receive advanced therapies but had no significant difference in mortality or length of stay compared with patients admitted directly through emergency departments [13]. These findings support PERT’s potential role in complex PE care, though prospective multicenter validation is needed.

The COVID-19 pandemic has added new dimensions to PE research [14]. While acute infection is a known prothrombotic trigger, the long-term influence of prior infection on PE is more complex [15,16]. In a recent multicenter study, nearly one-third of PE patients had a history of COVID-19, yet markers of embolism severity, such as RV/LV ratio > 1 on CT pulmonary angiography, were paradoxically more common in those without prior infection [17]. The absence of COVID-19 was also associated with higher odds of idiopathic PE, suggesting that post-COVID thrombotic risk may involve distinct mechanisms [17].

In cancer-associated VTE, the management of anticoagulation has shifted decisively toward DOAC-based strategies, anchored by randomized trials and now refined by new evidence for the extended phase. For initial 3–6-month treatment, apixaban, rivaroxaban, or edoxaban are appropriate for many patients, with apixaban shown to be noninferior to dalteparin without excess major bleeding in the CARAVAGGIO trial [18], while edoxaban and rivaroxaban reduce recurrence but require caution in luminal GI/GU cancers given the higher GI bleeding signals in Hokusai-VTE Cancer and early rivaroxaban data [19,20]. Contemporary guidance therefore favors DOACs for most patients without high bleeding risk or significant drug–drug interactions, reserving LMWH when bleeding risk is prominent (e.g., active GI lesions), interactions are problematic, or oral therapy is unsuitable [21]. The most notable update is API-CAT (2025): after the first 6 months, a reduced dose of apixaban at 2.5 mg twice daily was noninferior to a full dose (5 mg twice daily) in preventing recurrent VTE and reduced bleeding, supporting a step-down strategy for extended therapy in patients with active cancer who still warrant anticoagulation [22]. In practice, duration remains individualized—often continued while cancer is active or anticancer therapy ongoing—balancing recurrence risk, bleeding phenotype (especially GI/GU), renal function, and patient preferences, with LMWH as a viable alternative when DOACs are less suitable [23].

In unusual-site venous thrombosis, treatment is generally unified by prompt full-dose anticoagulation unless contraindicated [24]. Pragmatically, we should favor DOACs when bleeding risk and drug–drug interactions are low and absorption is reliable, and should prefer LMWH in pregnancy and in the presence of active GI lesions/bleeding, advanced liver disease, or significant interactions [24].

Prognostic research is also rapidly expanding. The AST/ALT (De Ritis) ratio, commonly used in hepatology, has been shown to predict adverse outcomes in PE [25]. Patients with elevated ratios (>1) were found to have nine-fold higher 60-day mortality, as well as increased risks of shock, vasopressor use, and extracorporeal life support [25]. Similarly, inflammatory markers such as the neutrophil-to-lymphocyte ratio (NLR) have been correlated with mortality and clinical severity, and when combined with PE severity index (PESI) or simplified PESI scores through machine learning models, have yielded refined risk categories with distinct mortality profiles [26]. These results highlight the promise of combining accessible biomarkers with computational tools to improve risk stratification.

A key question in secondary prevention after unprovoked VTE [27] is whether D-dimer-guided strategies can safely determine when to stop anticoagulation; the DULCIS and APIDULCIS management studies directly addressed this issue. The DULCIS study—pre-DOAC—used serial D-dimer testing with age/sex-specific cutoffs (and residual vein thrombosis assessment) to guide discontinuation: patients with persistently negative D-dimer who stopped anticoagulation had low recurrence (~3% patient-years), whereas those with positive tests who declined resumption had substantially higher recurrence; bleeding on resumed VKA therapy was non-trivial [28]. In contrast, APIDULCIS—in the DOAC era—embedded serial D-dimer into a strategy that gave a reduced-dose apixaban 2.5 mg bid only to patients with a positive test; the trial was halted early because outcomes were far worse in patients who stopped anticoagulation after a negative D-dimer (7.3%) than in those who received low-dose apixaban (1.1%; adjusted HR, ~8.2), leading to the conclusion that D-dimer alone should not be used to withhold extended therapy, while confirming the efficacy/safety of low-dose apixaban for extension [29]. Critically, both were nonrandomized management studies; differences in selection (anticoagulation ≥ 12 months prior to APIDULCIS), D-dimer assay/threshold heterogeneity, and contextual factors (e.g., COVID-19 pandemic era) likely contributed to divergent results, and in contemporary practice, a “stop-if-negative” approach appears unsafe, whereas extended low-dose DOAC is a more reliable default pending randomized data.

Device-based prevention strategies remain a controversial area. Inferior vena cava (IVC) filters, indicated in patients with contraindications for anticoagulation, are associated with long-term complications if not retrieved on time. Despite guideline recommendations, retrieval rates remain suboptimal. A recent multi-center retrospective analysis of more than 12,000 patients across 158 U.S. facilities revealed striking variability, with retrieval rates ranging from less than 1% to 100% and a mean of only 23% [30]. Moreover, only 43% of filters were retrieved within 90 days, the recommended timeframe [30]. High procedural volume did not correlate with better retrieval performance, and several high-volume centers demonstrated potential guideline non-adherence [30]. These findings highlight a systemic gap and the need for quality improvement initiatives to standardize retrieval practices and minimize complications.

Emerging factor XI/XIa inhibitors aim to decouple thrombosis from hemostasis and thus prevent VTE with less bleeding: the epidemiology of FXI deficiency and early clinical studies support this concept [31,32]. In phase 2 orthopedic prophylaxis, milvexian (oral FXIa inhibitor) and abelacimab (monoclonal anti-FXI) each reduced postoperative VTE with low bleeding versus enoxaparin, and an FXI antisense oligonucleotide similarly lowered VTE after knee arthroplasty [33,34,35]. Large phase 3 programs are now testing abelacimab for cancer-associated thrombosis (ASTER vs. apixaban; MAGNOLIA vs. dalteparin) [NCT05171049; NCT05171075], a population in whom a safer anticoagulant would be especially valuable; until these treatment trials are completed, DOACs remain the standard of care and FXI inhibitors should be viewed as promising but investigational.

Taken together, these studies reflect the rapidly evolving landscape of PE and VTE care. Diagnostics are becoming smarter and more resource-conscious, anticoagulation is more effective and individualized, interventional therapies are expanding but remain incompletely validated, and new biomarkers and computational tools are reshaping risk stratification [36]. Yet critical gaps remain. Randomized comparisons of invasive versus conservative therapy are urgently needed, as are strategies to personalize extended anticoagulation in high-risk populations such as cancer patients, those with obesity or renal impairment, and individuals with a history of COVID-19 [37]. Structured post-PE follow-up pathways to prevent chronic complications like chronic thromboembolic pulmonary hypertension (CTEPH) are lacking, and biomarkers such as the De Ritis ratio and NLR require multicenter validation. Addressing variability in device-related practices such as IVC filter retrieval is another crucial priority. The future of PE and VTE research lies in integrating these advances into cohesive, evidence-based care pathways. By uniting advances in diagnostics, therapeutics, prognostication, and healthcare delivery, the field has the opportunity to transform outcomes for patients with PE and VTE.
